# Dermoscopic characteristics of Merkel cell carcinoma

**DOI:** 10.1186/s12885-024-12566-2

**Published:** 2024-07-01

**Authors:** Dimitra Koumaki, Georgios Evangelou, Alexander C. Katoulis, Zoe Apalla, Aimilios Lallas, Marios Papadakis, Stamatios Gregoriou, Elizabeth Lazaridou, Konstantinos Krasagakis

**Affiliations:** 1 Department of Dermatology and Venereology, University Hospital of Heraklion, Heraklion, 71 500 Greece; 2 2nd Department of Dermatology and Venereology, Medical School, National and Kapodistrian University of Athens, “Attikon” General University Hospital, Rimini 1, Haidari, Athens, 124 62 Greece; 3 2nd Department of Dermatology, Medical School, Aristotle University of Thessaloniki, Papageorgiou General Hospital, Agiou Pavlou 76, Pavlos Melas, Thessaloniki, 564 29 Greece; 4 First Department of Dermatology and Venereology, School of Medicine, Aristotle University, 124 Delfon str, Thessaloniki, 54645 Greece; 5 Department of Surgery II, Witten/Herdecke University, Heusnerstrasse 40, 42283 Witten, Germany; 6 Department of Dermatology and Venereology, Andreas Sygros Hospital, Medical School of Athens, National and Kapodistrian University of Athens, I. Dragoumi 5, Athens, 161 21 Greece

**Keywords:** Dermoscopy, Reflectance confocal microscopy (RCM), Merkel cell carcinoma (MCC), Polymorphous vessels, Polyomavirus, Neuroendocrine carcinoma

## Abstract

**Background:**

Merkel cell carcinoma (MCC) is a rare, aggressive, cutaneous tumour with high mortality and frequently delayed diagnosis. Clinically, it often manifests as a rapidly growing erythematous to purple nodule usually located on the lower extremities or face and scalp of elderly patients. There is limited available data on the dermoscopic findings of MCC, and there are no specific features that can be used to definitively diagnose MCC.

**Aim of the study:**

Here, we aimed to summarize existing published literature on dermatoscopic and reflectance confocal microscopy (RCM) features of MCC.

**Materials and methods:**

To find relevant studies, we searched the PubMed and Scopus databases from inception to April 12, 2023. Our goal was to identify all pertinent research that had been written in English. The following search strategy was employed: (“ dermoscopy” OR “ dermatoscopy” OR “ videodermoscopy” OR “ videodermatoscopy” OR “ reflectance confocal microscopy”) AND “ Merkel cell carcinoma”. Two dermatologists, DK and GE, evaluated the titles and abstracts separately for eligibility. For inclusion, only works written in English were taken into account.

**Results:**

In total 16 articles were retrieved (68 cases). The main dermoscopic findings of MCC are a polymorphous vascular pattern including linear irregular, arborizing, glomerular, and dotted vessels on a milky red background, with shiny or non-shiny white areas. Pigmentation was lacking in all cases. The RCM images showed a thin and disarranged epidermis, and small hypo-reflective cells that resembled lymphocytes arranged in solid aggregates outlined by fibrous tissue in the dermis. Additionally, there were larger polymorphic hyper-reflective cells that likely represented highly proliferative cells.

**Conclusion:**

Dermoscopic findings of MCC may play a valuable role in evaluating MCC, aiding in the early detection and differentiation from other skin lesions. Further prospective case-control studies are needed to validate these results.

## Introduction

**Fig. 1:**
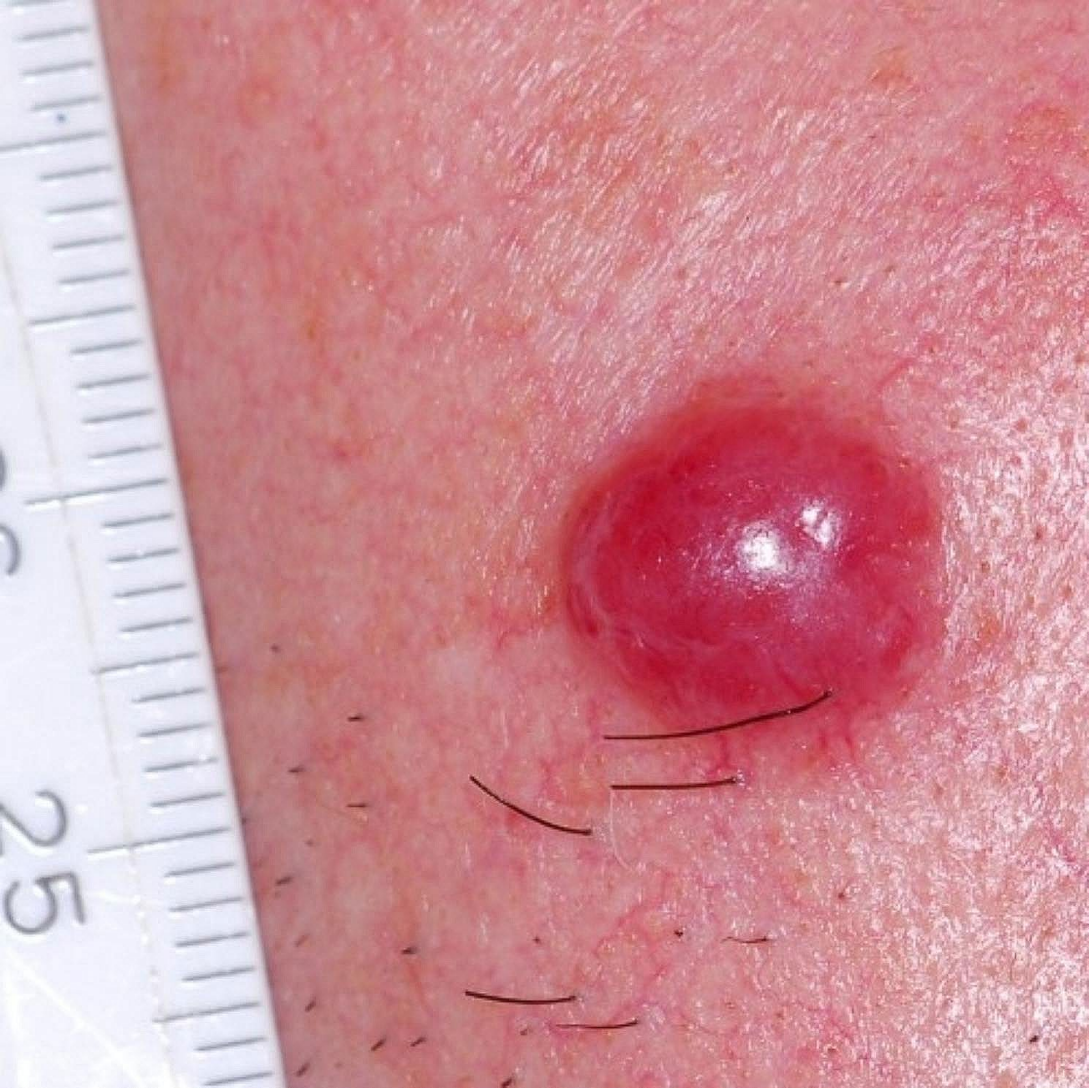
Clinical presentation of Merkel cell carcinoma presenting as a violaceous nodule

**Fig. 2:**
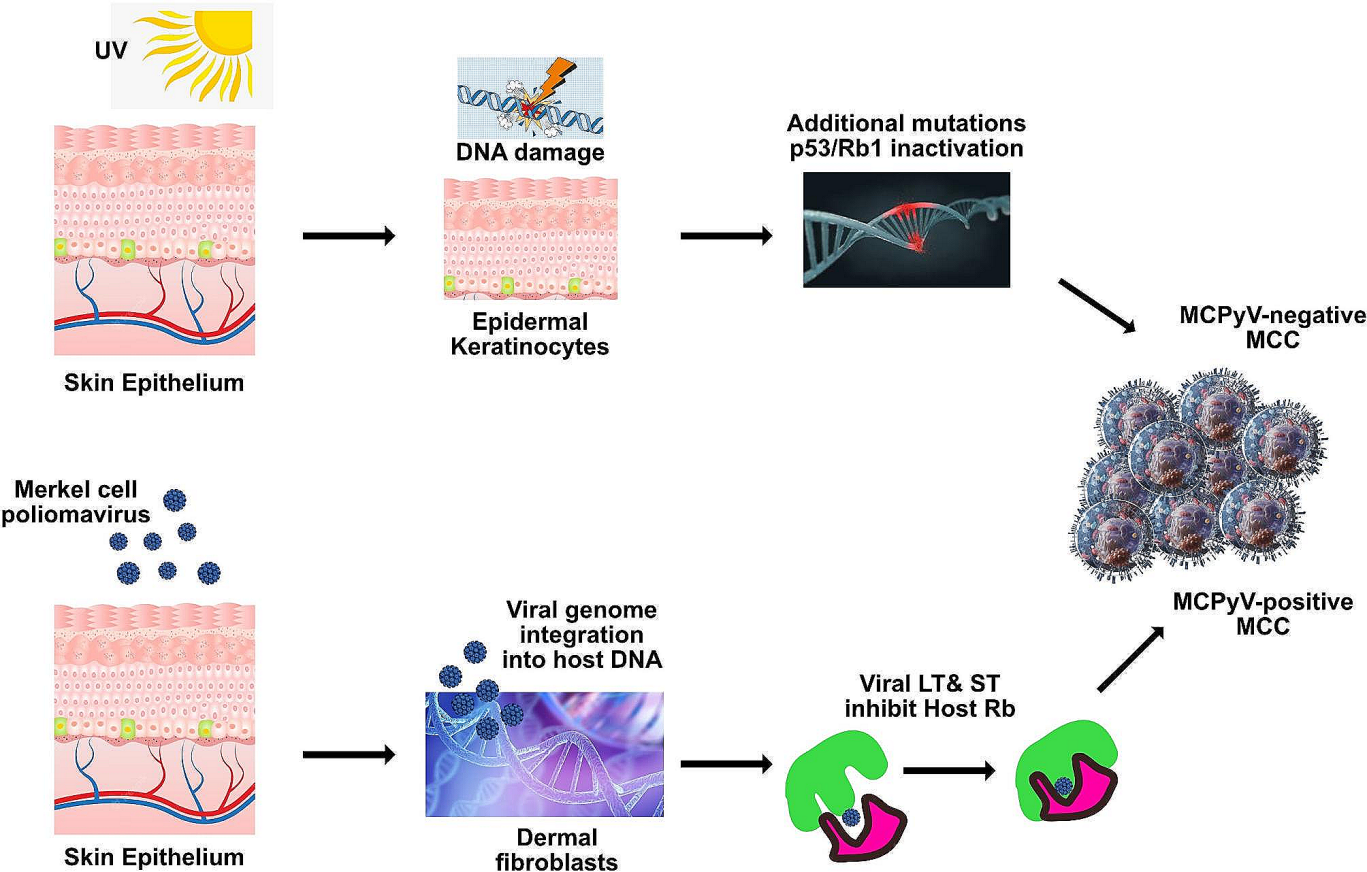
Pathogenesis of Merkel cell carcinoma. Adapted with permission from Hernandez LE, et al. *Dermatol Ther*. 2022 Mar;35(3):e15292. doi: 10.1111/dth.15292

Merkel cell carcinoma (MCC) is a rare but aggressive type of skin cancer that typically affects older individuals with fair skin. It usually presents as a rapidly growing, firm nodule that may be flesh-colored or bluish-red in appearance, often found in sun-exposed areas of the body (Fig. 1) [1]. Recent studies from various countries have reported an increasing incidence of MCC over the past few decades [2–5]. Based on early histologic and ultrastructural studies, it is believed that MCC originates from Merkel cells, which are found in the basal layer of the epidermis and hair follicles and are associated with sensory neuritis in the dermal papillae, the skin mechanoreceptors [6]. However, this hypothesis is controversial, and an alternative hypothesis suggests that these tumors may originate from immature multipotent stem cells that acquire neuroendocrine features during malignancy transformation (Fig. 2). UV radiation exposure may be particularly relevant in the pathogenesis of the virus-negative subtype of MCC, as evidenced by a lower prevalence of MCPyV-positive tumours in areas with sun damage [6].

There is limited available data on the Dermoscopic reflectance confocal microscopy (RCM) findings of MCC, and there are no specific features that can be used to definitively diagnose MCC. In this study, we aimed to summarize existing published data on the dermoscopic and RCM features of MCC.

## Materials and methods

The Preferred Reporting Items for Systematic Reviews and Meta-Analyses (PRISMA) standards were followed for conducting this systematic review with meta-analysis [7]. To find relevant studies, we searched the PubMed and Scopus databases from inception to April 12, 2023. Our goal was to identify all pertinent research that had been written in English. The following search strategy was employed: (“ dermoscopy” OR “ dermatoscopy” OR “ videodermoscopy” OR “ videodermatoscopy” OR “ reflectance confocal microscopy”) AND “ Merkel cell carcinoma”. Two dermatologists, DK and GE, evaluated the titles and abstracts separately for eligibility. For inclusion, only works written in English were taken into account.

All articles detailing the dermoscopic characteristics of MCC in one or more patients were considered. Reference lists of papers that were included were checked again for more publications that qualified. A consensus was reached in the event of a disagreement between the two researchers. The clinical, dermoscopic, RCM and histological data were collected from each relevant article after it had been fully retrieved. Based on the initial description provided by the authors of the included studies, the dermoscopic characteristics of MCC were initially evaluated.

## Results

**Fig. 3:**
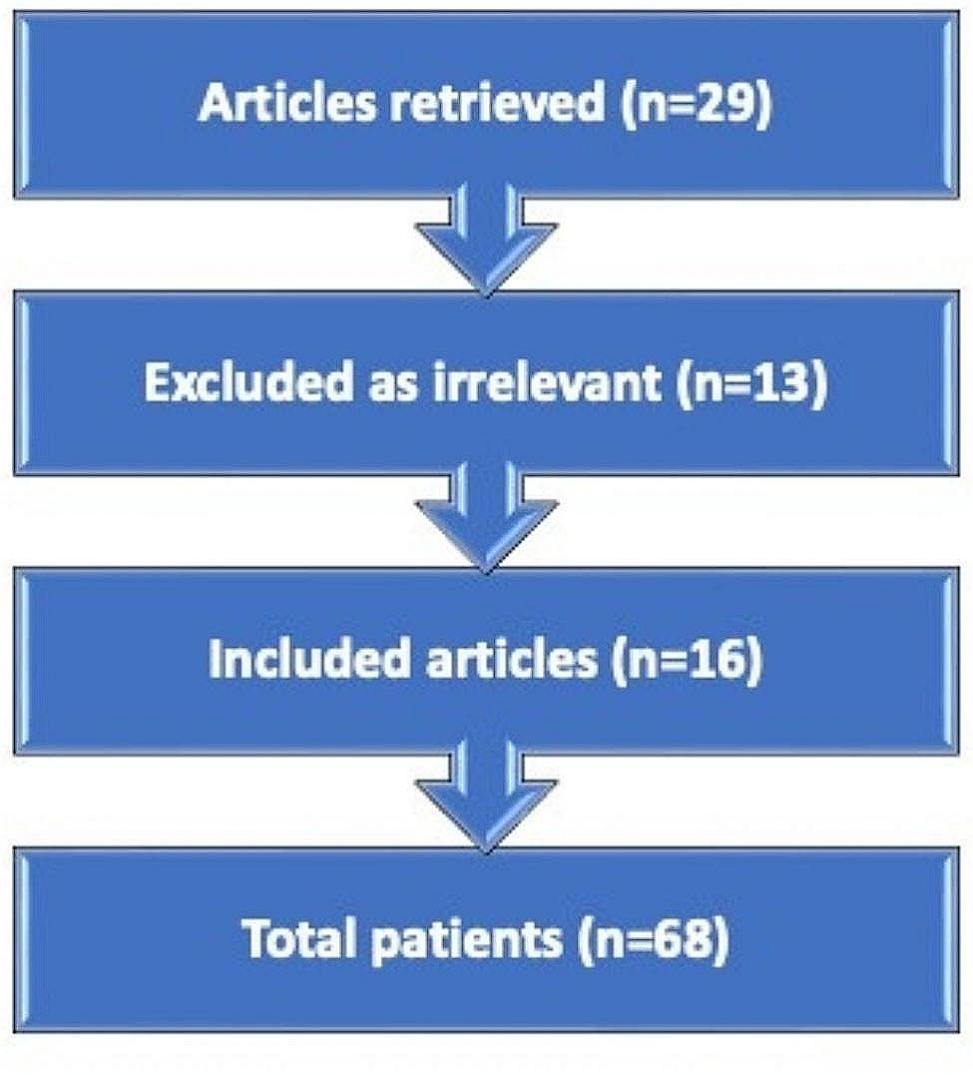
Flow diagram

**Fig. 4:**
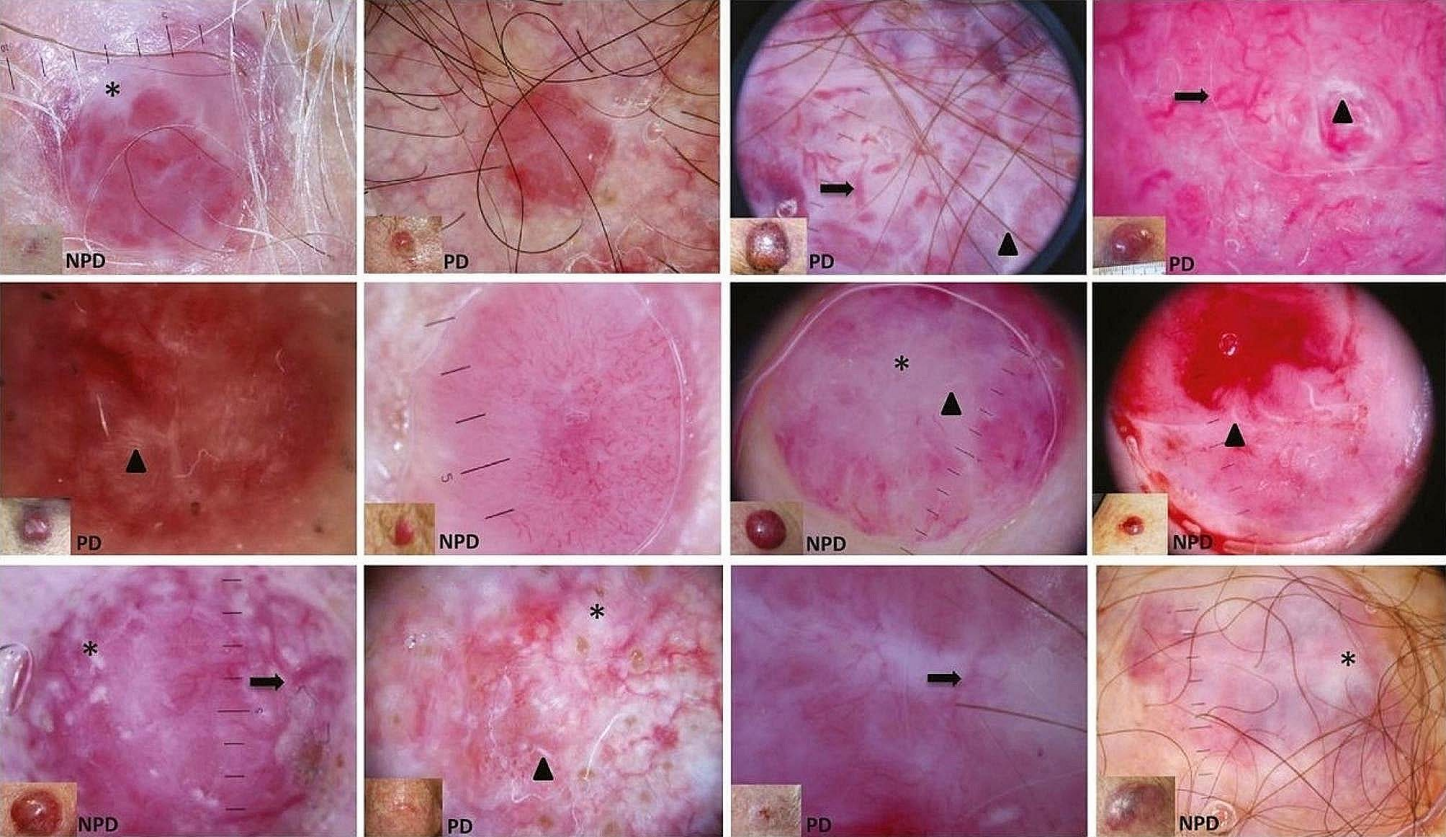
Clinical and dermoscopic images of patients with Merkel cell carcinoma (MCC). Some dermoscopic characteristics are marked as follows: arrows: large vessels, arrowheads: shiny white areas, asterisk: non-shiny white areas. NPD: non-polarized dermoscopy, PD: polarized dermoscopy. Reproduced with permission from Jalilian C. et al. *Br J Dermatol*. 2013 Aug;169(2):294-7. doi: 10.1111/bjd.12376

In total 29 articles were retrieved of which 13 were irrelevant, therefore 16 articles were included in this study, in a total of 68 MCC cases (Fig. 3). The dermoscopic and RCM characteristics of MCC cases of our study are summarized in Table 1 [8–23]. The most common dermatoscopic signs included the following (Fig. 4):

**Table 1:** Summary of dermatoscopic characteristics of previously published Merkel cell carcinoma (MCC) cases

		Architecture	Epidermal component			Dermal (stromal) component							Vascular component										
													Vessel location										
													Deep vessels	Superficial vessels									
			**Homogenous**	**Periphery**	**Ulceration**	**Fibrous component**				**Microvascular component**					**Size**		**Vessel morphology**						
**Author’s name, year of publication**	**Number of cases**	**Architectural disorder**	**Scale**	**Collaret**		**Structureless areas**	**Horseshoe-like structures**	**White areas** **(Milky white background)**	**Shiny white lines**	**Pinkish structureless background**	**Purpuric red to violet background**	**Milky red areas**	**Poorly focused vessels**	**Sharply focused vessels**	**Large vessels**	**Dotted vessels**	**Polymorphous vessels**	**Linear irregular vessels**	**Linear beaded vessels**	**Arborizing vessels**	**Glomerular vessels**	**Comma vessels**	**Lacunes/** **Vascular lakes**
Navarrete-Dechent, et al. 2020 [21]	1		Yes 1/1						Yes 1/1	Yes 1/1						Yes 1/1							
Sadeghinia et al. 2019 [8]	1		Yes 1/1 (100%)			Yes 1/1 (100%)	Yes	Yes 1/1 (100%)				Yes 1/1 (100%)			Yes 1/1 (100%)	Yes 1/1 (100%)	Yes 1/1 (100%)	Yes 1/1 (100%)	Yes	Yes 1/1 (100%)			
Lin MJ et al. 2019 [9]	6		2/6 (33%)		2/6 (33%)				5/6 (83%)			5/6 (83%)		6/6 (100%)			5/6 (83%)	5/6 (83%)		6/6 (100%)			2/6 (33%)
Cinotti E. et al. 2019 [23]	4 patients with primary tumours of MCC									4/4 (100%)								Yes 4/4 (100%)		Yes 2/4 (50%)			
	1 of 4 patients had 6 lesions of metastatic MCC on the same limb (in total 10 lesions in this article)							Yes (whitish areas) 6/6 lesions (100%) on the same patient			Yes 6/6 lesions (100%) on the same patient							6/6 (100%)		3/6 (50%)			
Geller S., et al. 2017 [10]	1							Yes 1/1 (100%) With a diffuse milky white background										Yes 1/1 (100%)					
Longo C. et al. 2017 [22]	1										1/1 (100%) With crystalline structures							1/1 (100%)					
Di Meo N. et al. 2017 [11]	1										1/1 Reddish background							Rainbow-patterned areas surrounded by reddish background, ectatic vessels					
Suarez AL, et al. 2015 [12]	9 SCC/MCC		9/9 (100%)									In some cases milky red areas located centrally			In some cases	9/9 (100%)		9/9 (100%) Short linear irregular vessels at the periphery		In some cases			
Laureano, et al. 2014 [13]	1							1/1 (100%)	1/1 (100%)			1/1 (100%)	1/1 (100%)	1/1 (100%)	1/1 (100%)		1/1 (100%)	1/1 (100%)					
Lallas A. et al. 2014 [14]	6											6/6 (100%)				6/6 (100%)	6/6 (100%)	6/6 (100%)		6/6 (100%)	6/6 (100%)		
Scalvenzi M. et al. 2013 [15]	1											1/1 (100%)					1/1 (100%)						
Jalilian C., et al. 2013 [16]	12	9/12 (75%)		1/12 (8%)		12/12 (100%)		11/12 (92%) Non shiny white areas 9/12 (75%) Shiny white areas				11/12 (92%) Milky pink/red areas or globules	12/12 (100%)	8/12 (67%)	9/12 (75%)	1/12 (8%)	12/12 (100%)	12/12 (100%)		7/12 (58%)			
Gontijo B. et al. 2012 [17]	1									1/1 (100%) Homogeneous pink background							1/1 (100%)	1/1 (100%)				1/1 (100%)	
Dalle S, et al. 2012 [18]	10											8/10 (80%)				4/10 (40%)		4/10 (40%)		4/10 (40%)	1/10 (10%)		
Harting MS, et al. 2011 [19]	10		2/10 (20%) Surfaces scale			0/10						Yes (10/10, 100%) Milky red areas/globules				3/10 (30%) Small dotted vessels		Yes (10/10, 100%)		6/10 (60%) These are located depper within the tumour and consequently are slightly out-of-focus and pink in color	3/10 (30%)		
Ciudad C, et al. 2010 [20]	2						1/1 (100%)											1/1 (100%)				1/1 (100%)	
							1/1 (100%)			1/1 (100%) Homogeneous pinkish background								1/1 (100%)				1/1 (100%)	

### Milky red areas

On dermoscopy, milky red areas were reported in 49/68 (72.05%) of published MCC cases. Milky red areas, also called pink-white structureless areas, and creamy red background or pink homogeneous areas, refer to structureless areas exhibiting pink to-white-red colour [24–26]. Although the occurrence of milky red areas in skin lesions is infrequent, their presence has been strongly linked to invasive melanoma. These areas/globules may help identify thicker amelanotic/ hypomelanotic melanomas (AHM), which often lack traditional melanoma features [27–29]. In AHM, milky read globules/areas were observed in 31% of thin melanomas (1 mm) and 93.3% of thick melanomas, in contrast to 17.3% and 9.1% of amelanotic/hypomelanotic benign melanocytic lesions and amelanotic/hypomelanotic non-melanocytic lesions, respectively, according to a study [30, 31]. Vascular blush, also known as erythematous blush, pink veil, or milky red patches, is a red or pink area, corresponding to a more vascularized site of a lesion. Vascular blush can therefore be seen in a wide range of dermatologic entities, including dermatofibromas, vascular tumours such as pyogenic granulomas (PG), inflammatory lesions, melanoma, and nonmelanoma skin malignancies. Regarding melanocytic lesions, vascular blush can be seen in nevi, but it is more frequent and noticeable in melanoma [32, 33].

### Linear irregular vessels

**Fig. 5:**
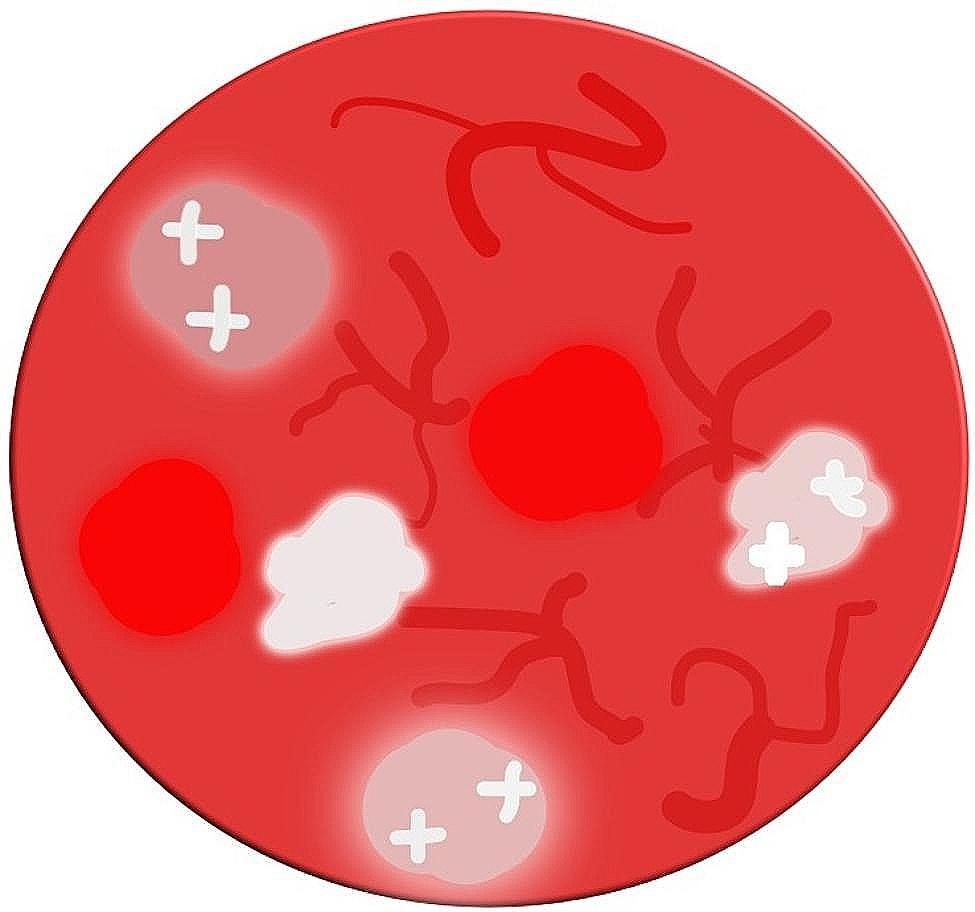
Schematic approach of dermoscopic characteristics of Merkel cell carcinoma (MCC) indicating multiple arborizing irregular, polymorphous vessels, shiny white, milky red areas as well as structureless areas Adapted with permission from Jalilian C. et al. *Br J Dermatol.* 2013 Aug;169(2):294-7. doi: 10.1111/bjd.12376

Linear irregular vessels were noticed under dermoscopy in MCC cases with a frequency of 64/68 (94.11%) (Fig. 5). These vessels are red vascular structures that vary in size, shape, and distribution. The presence of irregular vessels is a strong indication of malignancy since they are commonly found in melanoma, basal cell carcinoma (BCC), and squamous cell carcinoma (SCC). In a previous study, linear irregular vessels were identified in 33.3% of melanomas, with a positive predictive value (PPV) of 67.6% [30].

### Arborizing vessels

The presence of arborizing vessels was reported in 51/68 (75%) of cases (Figs. 4 and 5). Arborizing vessels are vessels with large diameter, branching irregularly into fine capillaries. They are usually seen in BCC but they can also be present in furuncles, cysts, intradermal nevi, and other adnexal tumours. Bright-red, in focus, arborizing vessels have a PPV of 94% for BCC [34–36]. Arborizing vessels, when viewed under dermoscopy, are red linear vessels with a thick diameter (0.2 mm or more) that split into branches with progressively thinner diameters, resembling a tree-like pattern. In BCC these vessels are sharply focused due to their superficial location just below the epidermis and exhibit irregular branching into small diameter capillaries (10 μm). Traditionally, arborizing vessels were considered a reliable indicator of BCC, with a PPV of 94%, sensitivity of 96,1%, and specificity of 90.9%. However, a recent study from Korea found that only 54% of lesions displaying arborizing vessels (Avs) were confirmed as BCCs. The variation in PPV for BCC diagnosis using arborizing vessels can be attributed to the types of lesions included in the analysis. When only lesions in the differential diagnosis for BCC are considered, the PPV of arborizing vessels may be higher compared to when all lesions, regardless of differential diagnosis, are included. It is worth noting that adnexal tumours can sometimes exhibit similar features, including arborizing vessels, as BCCs [34, 35].

### Polymorphous vessels

Polymorphous vessels were seen in 27/68 (39.70%) of MCC cases (Figs. 4 and 5). Polymorphous vessels are a combination of two or more vessel morphologies, with the most common combination being dotted and serpentine vessels. Polymorphous vessels, corresponding to the neovascularization of a tumour, have been traditionally considered an indicator of melanoma. A study of lesions with vascular features on dermoscopy showed that only 20% of the lesions had a polymorphous pattern and their presence had a PPV of 52.6% for melanoma. Specifically, the combination of dotted and linear-irregular vessels was found in 18% of amelanotic/ hypomelanotic melanomas, but rarely in other nonmelanoma amelanotic/hypomelanotic lesions [36, 37]. Polymorphous vessels may also be present in cutaneous metastases, and other tumours such as eccrine poromas [29].

### Lacunes/vascular lakes

Lacunae were observed in 2/68% of MCC cases. Lacunae are clusters of numerous well-defined round or oval, reddish formations that are divided by septae, a white rim. They are comparable to dilated, thin-walled veins in the papillary dermis histologically. Vascular tumours, especially angiomas, are distinguished by lacunae. Black (violaceous, blue-black, or black) lacunae that are extremely distinctive to isolated angiokeratomas show partially or entirely thrombosed dermal arteries lying deeper in the dermis.

### Ulceration

Ulceration was reported in 2/68 (2.94%) of MCC cases.

### Structureless areas

Structureless areas were described in 13/68 (19.11%) MCC cases. Regions within a lesion that lack any discernible feature or structure are referred to as “structureless areas”. These areas should occupy at least 10% of the total surface of the lesion. Structureless areas can exhibit hypopigmentation, hyperpigmentation, or regular pigmentation [37].

### Sharply focused vessels

Sharply-focused vessels were mentioned in 15/68 (22.05%) of MCC cases. Sharply focused vessels have been previously described in a case series of necrobiosis lipoidica (NL) [38].

### White areas (Milky white background)

White areas or milky white backgrounds were noticed in 20/68 (29.41%) of cases.

### Architectural disorder

Architectural disorder was reported in 9/68 (13.23%) of the cases.

### Dotted vessels

Dotted vessels were seen in 25/68 (36.76%) of MCC cases. Dotted vessels are small red dots, ranging in diameter from 0.01 to 0.02 mm that histologically correspond to vessels with perpendicular orientation to the skin surface. Dotted vessels can be observed in inflamed, traumatized, or stasis skin, as well as in cutaneous tumours. The presence of dotted vessels is often indicative of melanocytic lesions, with a study showing that 90% of lesions with dotted vessels were melanocytic. In melanocytic tumours, dotted vessels have varying PPV for different types of melanoma, ranging from 38% for melanoma to 16% for dermal/congenital nevi, 21% for Clark nevi, and 16% for Spitz nevi. In benign nevi, dotted vessels correspond to vessels at the tips of dermal papillae and are often observed within the pigment network. In melanoma, dotted vessels, usually found in conjunction with other types of vessels, can be located anywhere within the lesion but are more concentrated toward the center. Studies have shown that dotted vessels are commonly seen in thinner melanomas (< 1 mm), compared to thicker ones (> 1 mm), particularly in amelanotic and hypomelanotic melanomas [39–41].

### Large vessels

Large vessels were observed in 16 out of 68 cases (23.52%).

### Collarette

A collarette was shown only in one out of 68 cases (1.47%). A collarette refers to a thin rim of loosened keratin that overhangs the periphery of the skin lesion and is connected to the adjacent healthy skin. The external edge of the collarette is attached, whereas the internal edge is free. Collarette can be seen in eccrine poroma [28].

### Scale

Scale was described in 14/68 (20.58%) cases.

### Glomerular vessels

Glomerular vessels were noticed in 10/68 (14.70%) of cases. Glomerular vessels are coiled vessels mimicking the glomerular apparatus of the kidney. They can be seen in Bowenoid actinic keratosis, Bowen’s Disease (BD), SCC, and clear cell acanthoma. They have a 62% PPV for SCC [37].

### Comma vessels

Comma vessels were reported in 3/68 (4.41%) cases. Comma vessels which are vessels that are curved in a shape resembling a comma are typically blurry when viewed under dermoscopy due to their deeper location within the dermis. These vessels are commonly associated with dermal naevi, with studies showing that 66.3% of dermal/ congenital naevi exhibit this vascular pattern, and they have a PPV of 94% [36, 37]. Additionally, in another study, the presence of organized comma vessels as the predominant vessel morphology is indicative that the lesion is not a melanoma, suggesting a benign naevus, commonly dermal or compound naevus.

### Horseshoe-like structures

Horseshoe-like structures were described in 3/68 (4.41%) of cases.

### Linear beaded vessels

Linear beaded vessels were seen in 9/68 (13.23%) of cases.

### Pinkish structureless background

Pinkish structureless background was observed in 7/68 (10.29%) of cases.

### Shiny white lines

Shiny white lines were reported in one case 6/68 (8.82%). These white streaks are oriented in parallel or sometimes perpendicular to each other. In dermoscopy, perpendicular white lines are short, separate white lines that are oriented parallel or perpendicular to each other and can only be seen under polarized light. They are also known by various names such as polarizing white lines, short white lines, shiny white lines, whiny white streaks, chrysalis, chrysalids, and crystalline structures. Polarized dermoscopy enables improved visualization of deep structures and reveals structures that are not visible with non-polarized dermoscopy, such as 4-dot clods (also known as rosettes) and shiny bright, linear, and white lines referred to as shiny white streaks (SWSs). SWSs can be found in both benign and malignant skin lesions, including melanoma, Spitz naevus, dysplastic naevus, intradermal naevus, BCC, dermatofibroma, scar tissue, and benign lichenoid keratosis. Previous studies have shown that SWSs are more prevalent in melanomas (ranging from 23.4 to 32.8%) compared to melanocytic naevi (ranging from 0.07 to 2.6%) and their visualization as thin, shiny, linear, white structures depends on the use of polarized dermoscopy. The presence of SWSs histologically corresponds to broad deep dermal fibroplasia. Cases with diffuse SWSs exhibited a significantly higher degree of deep fibroplasia than cases with focal SWSs [41, 42].

### Reflectance confocal microscopy (RCM) findings in MCC

RCM is a non-invasive technique that allows for high-resolution imaging that can provide detailed images of the cellular and structural features of the skin, including the epidermis, dermis, and other skin layers, as well as blood vessels and hair follicles [43, 44].

In the case presented by Longo et al., which was a primary MCC, the use of reflectance confocal microscopy (RCM) showed that there were clusters of small cells that appeared darker compared to lymphocytes, and they were larger than normal cells. Some of these cells had irregularly shaped nuclei that filled up the entire cell (Table 2). These clusters were surrounded by fibrotic tissue. In live imaging, large blood vessels were observed near the tumour growth. Additionally, within these dark cells, some bright cells likely represented highly proliferative cells, which appeared more reflective in RCM [22].

**Table 2:** Summary of reflectance confocal microscopy (RCM) findings of Merkel cell carcinoma (MCC) cases of the existing published literature

Author, Year of publication	Country	Journal	Number of cases	Gender M/F	Age	Area affected	Clinical presentation	Duration of lesion	Type of dermatoscope / Confocal Microscopy (CM) used	RCM Dark pagetoid cells in the epidermis	RCM Epidermis: Thin and disarranged by the underlying tumours	RCM, Glandular nests at the dermoepidermal junctions separated by septae	RCM Dissociation of cell aggregates	RCM Dermis: Small hyporeflective cells resembling lymphocytes arranged to form solid aggregates outlined by connective fibrous tissue	RCM There were some larger polymorphic hyper-reflective cells	RCM Some cells revealed large and pleomorphic nuclei that occupied completely the cell	RCM Large calibre vessels were observed in proximity to tumours’ proliferation	RCM Within the hyporefrective cells some bright cells were also observed
Navarrete-Dechent, et al., 2020 [21]	Santiago, Chile and New York, USA	Aust J Derm	1	M	60	Right eyebrow	Asymptomatic erythematous scaly plaque	3 years	- RCM (VivaScope 3000, Caliber Imaging and Diagnostics Rochester, NY, USA)	Yes 1/1		Yes 1/1						
Cinotti E. et al., 2019 [23]	St-Etienne, France and Siena, Italy	Skin Res Technology	4 patients with primary tumours of MCC	2 males and 2 females	Mean age = 72-year-old (range 68–75)	50% lower limbs, 50% head	Rapidly growing, asymptomatic, firm, erythematous plaques.		PowerShot G7 camera (Canon, New York, USA), combined with FotoFinder Systems GmbH, Bad Birnbach, Germany x 20 magnification. Reflectance Confocal Microscopy (RCM)		Yes 4/4 (100%)		Yes 4/4 (100%)	Yes 4/4 (100%)	Yes 4/4 (100%)			
			1 of the 4 patients with had also 6 lesions of metastatic MCC on the same limb			All lesions were located on the same leg					Yes 6/6 (100%)		Yes 6/6 (100%)	Yes 6/6 (100%)	Yes 6/6 (100%)			
Longo C. et al., 2017 [22]	Modena, Reggio Emilia, Italy	JEADV	1	M	86	Left lower limb	Large red and fast-growing nodule 3 × 2 cm							1/1 (100%)		1/1 (100%)	1/1 (100%)	1/1 (100%)

*Abbreviations* RCM: reflectance confocal microscopy, MCC: Merkel cell carcinoma

Cinotti et al. described four patients who had four primary MCC lesions and one of them had six lesions of metastatic MCC located on the same limb. The RCM features observed in all cases by Cinotti et al., were very similar and confirmed the morphological aspects described in a previous study by Longo et al. The RCM images showed small hypo-reflective cells that resembled lymphocytes arranged in solid aggregates outlined by fibrous tissue in the dermis. Additionally, there were larger polymorphic hyper-reflective cells that likely represented highly proliferative cells. The epidermis appeared thin and disarranged due to the underlying tumours. Metastatic lesions exhibited the same RCM features. While these findings could also be seen in cases of amelanotic melanoma, MCC showed a less pronounced polymorphous appearance and a more evident fibrotic stroma between the cell aggregates, which supported the diagnosis of a non-melanocytic skin tumour. Notably, apart from the findings previously reported in the RCM case described earlier by Longo et al., Cinotti also noted the dissociation of cell aggregates in certain regions of MCC. While this dissociation has been suggested to be a potential artifact resulting from tissue processing and fixation, it should not be dismissed as such, as it was consistently observed in vivo in RCM images and was in perfect alignment with histopathology findings, similar to clefts around to BCC [23].

In a case of intraepidermal MCC described by Navarrette-Dechent et al., RCM assessment using VivaScope 3000, a device by Caliber Imaging and Diagnostics in Rochester, NY, USA, revealed the presence of dark pagetoid cells in the epidermis, as well as glandular nests at the dermo-epidermal junction that were separated by septae [21].

## Discussion

One of the characteristic, though non-pathognomonic, dermoscopic findings in MCC is the presence of polymorphous vessels, which are typically irregular or tortuous, and neoangiogenesis, crucial for tumor growth and invasion, may be associated with these dermoscopic structures in MCC [8–23].

These vessels can appear as arborizing or linear with irregular shapes and sizes and are often distributed in a scattered or clustered pattern. Arborizing vessels, typically seen in BCC, are clearly defined with a distinct red color, indicating their superficial location. In contrast, the poorly focused and pink-colored vessels observed in MCC suggest a deeper vessel location. Histopathological findings support the notion that deep angiogenesis is associated with milky red areas in dermoscopy [24–26]. Well-defined large arborizing vessels may correspond to new vessels located closer to the surface, while blurry vessels may be located deeper. Linear-irregular vessels may indicate elongation of capillaries and tortuous horizontal vessels [8–12]. Milky-red areas or globules may also suggest deep tumoral angiogenesis, which could explain the presence of blurred white and pink structures. The disordered formation of new blood vessels, a characteristic feature of MCC, may account for the appearance of the rainbow pattern observed in such lesions. This pattern, typically seen in Kaposi sarcoma, could aid in diagnosing MCC [24].

One study demonstrated a link between the irregular linear vessels observed through dermoscopy and the presence of clustered dilated vessels at the tumor’s periphery upon histopathological examination [35]. These linear irregular vessels are indicative of small, structurally abnormal, and variably dense blood vessels, commonly seen in tumor angiogenesis. They often occur in areas where vessel dilation is prominent. Linear irregular vessels and milky red areas, although common in hypomelanotic and amelanotic melanoma, are less distinctive for MCC. Additionally, the clustering of dilated blood vessels in the middle dermis, along with increased vascular proliferation in the deeper dermis, can correspond to irregular vessels and milky red areas observed in dermoscopy. Another characteristic dermoscopic feature, known as glomerular vessels, is frequently seen in Bowen’s disease (BD) [ 25, 26]. The significance of shiny white chrysalis-like structures in MCC is not yet understood.

The differential diagnoses often considered alongside MCC include amelanotic melanoma, BCC, SCC, cutaneous B-cell lymphoma, and PG [14–16 ]. We observed that MCC typically lacks pigmented structures or a blue-grey veil, which helps distinguish it from hypomelanotic melanoma. Unlike well-differentiated SCCs, MCCs generally lack hyperkeratosis. The presence of a collarette is infrequent in MCCs, which is an important clue for distinguishing MCC from PG, which often exhibits milky red areas. Scale and hyperkeratosis, also present in BD and SCC, may suggest epidermotropism when found in MCC, as evidenced by a case of combined MCC/SCC [12].

RCM is a supplementary diagnostic technique that enables in-depth examination of the skin at a resolution close to that of histology. Reported RCM findings of MCC included small hypo-reflective cells forming solid aggregates in the presence of fibrotic stroma and prominent vasculature. These findings were similar to some melanoma subtypes (such as nevoid melanoma), but the monomorphous appearance favored a diagnosis of non-melanoma skin cancer, specifically not BCC [ 39, 40 ].

## Conclusion

In conclusion, the dermoscopic findings of MCC are indicative of the rapid growth of this tumor. However, they are not specific to MCC, and therefore, their usefulness and value in diagnosis and management are unknown. All studies were descriptive and observational, without evaluation of diagnostic significance.

## Data Availability

The data that support the findings of this study are available upon reasonable request from the corresponding author, MP.

## Abbreviations

MCC: Merkel cell carcinoma
PPV: Positive predictive value
SWSs: Shiny white streaks
BCC: Basal cell carcinoma
BD: Bowen’s Disease
SCC: Squamous cell carcinoma
RCM: Reflectance confocal microscopy
PG: Pyogenic granuloma
